# Lunar and Martian gravity alter immune cell interactions with endothelia in parabolic flight

**DOI:** 10.1038/s41526-024-00456-7

**Published:** 2025-02-03

**Authors:** Yu Du, Bing Han, Katharina Biere, Nathalie Abdelmalek, Xinyu Shu, Chaoyang Song, Guangyao Chen, Ning Li, Marina Tuschen, Huan Wu, Shujin Sun, Alexander Choukér, Mian Long, Dominique Moser

**Affiliations:** 1 Key Laboratory of Microgravity (National Microgravity Laboratory), Center of Biomechanics and Bioengineering, and Beijing Key Laboratory of Engineered Construction and Mechanobiology, Institute of Mechanics, Chinese Academy of Sciences, 100190 Beijing, China; 2 School of Engineering Science, University of Chinese Academy of Sciences, Beijing, China; 3 Laboratory of Translational Research ‘Stress and Immunity’, Department of Anesthesiology, LMU Hospital, Ludwig-Maximilians-University Munich, Munich, Germany

**Keywords:** Inflammation, Diseases

## Abstract

Returning to the moon and traveling to Mars represent the main targets of human space exploration missions within the upcoming decades. Comparable to microgravity, partial gravity in these destinations is assumed to dysregulate immune functions, thereby threatening astronauts´ health. To investigate the impact of partial gravity on immune cell attachment to vessel endothelia, THP-1 cells and HUVEC cell layers were monitored in a flow chamber system during parabolic flight in lunar (0.16 *g*) or Martian (0.38 *g*) gravity. Focus was set on floating speed, cell adhesion, surface molecule expression and cytoskeletal reorganization under basal and TNF-induced inflammatory environment. Floating speed of THP-1 cells was increased in partial gravity, which was accompanied by a successively lower adhesion to the endothelial HUVEC cells. Expression levels of the adhesion markers Mac-1 on THP-1 cells as well as ICAM-1 on HUVECs were found elevated in lunar and Martian gravity, which was aggravated by TNF. Analysis of cytoskeletal organization in HUVECs revealed reduced intracellular F-actin microfilament networks and a stronger cell directionality with stress fiber alignment at cell borders in partial gravity, which was intensified by TNF. In summary, altered immune cell - endothelium interactions as quantified in partial gravity conditions show similarities to cellular behavior in microgravity. However, the different magnitudes of effects in dependence of gravitational level still need to be assessed in further investigations.

## Introduction

Presently, moon and Mars constitute the primary targets of human space explorations, followed by building scientific research stations including human habitats. The flights to space of hundreds of astronauts since 1961 have shown that the human body encounters significant changes of the immune system^1^ that may require a suit of countermeasures^2,3^. The altered immune functions might increase the risk of infection, which coincides with reports that over 50% of the Apollo astronauts had bacterial or viral infections during spaceflight or within one week after return back to Earth^4^.

Many efforts have been put into studying the influence of microgravity (µ*g*) on the immune system either by real µ*g* in space stations and parabolic flights, or by simulating microgravity effects on Earth with designated devices, such as 2D clinostats, random positioning machines, rotational wall vessels, and magnetic levitation^5^, however, the effects of lunar and Martian gravity, which is referred to as partial or fractional gravity, are understudied by now. It is anticipated that physiological deconditioning in partial gravity might be less severe than in µ*g*, however, this assumption is based on linear regression analyses rather than on comparative experiments^6,7^. Since 2011, the induction of lunar and Martian gravity in Earth-bound models can be realized in parabolic flight by adjusting the injection angle to 40° for lunar and 30° for Martian gravity respectively. Despite the short duration of partial gravity (24 s in lunar gravity and 33 s in Martian gravity), parabolic flights represent a suitable platform for research under this exceptional condition.

A proper host immune cascade consists of circulatory immune cells´ rolling, adhesion, crawling and transmigration on vascular endothelia through interactions of adhesion molecules, including selectins, integrins and the CD44 glycoprotein^8^. Previous experiments in 2D clinostats, parabolic flights and suborbital flights have shown that µ*g* can affect the expression of adhesion receptors and ligands^9,10^. Monocytes increase their ICAM-1 expression but display decreased expression levels of L-selectin and a constant CD18 expression^11^. However, results vary between species and tissue-type. Moreover, we demonstrated in a parabolic flight certified and customized flow-chamber system an increased rolling speed of peripheral blood mononuclear cells (PBMCs) over an adhesion molecule substrate in parabolic flight-induced µ*g*, which was accompanied by a reduced rolling rate^12^. Together with altered expression patterns of adhesion molecules, this may inhibit transendothelial migration. In addition, alterations in gravity can also affect both the immune cell and endothelial cell cytoskeleton, which plays a key role in mechanical sensing and regulating cell behaviors in terms of adhesion, migration and inflammation.

Within the study presented herein we translated our flow chamber setting to fractional Earth gravity conditions reflecting the future exploration destinations. Here, we summarize the results of recruiting floating THP-1 monocytic cells to an endothelial cell layer in terms of floating speed, adhesion numbers, expression of adhesion molecules, and cytoskeleton organization. The results quantify that partial gravity affects both elements of immune cell recruitment in normal and inflammatory environments.

## Materials & methods

### Cell culture and glycocalyx production by HUVECs

For flow chamber experiments, human monocyte-like cell THP-1 cells were used as suspension cells. Human umbilical cord vein cells (HUVEC) were cultivated to create an endothelial cell layer within the flow chamber. THP-1 cells (cat. no. TIB-202, ATCC, Manassas, VA, USA) were cultured in RPMI-1640 medium (cat. no. 21875-034, Thermo Fisher, Waltham, MA, USA) supplemented with 10% (v/v) Fetal Bovine Serum (cat. no. P30-3306, Pan Biotech, Aidenbach, Germany), Penicillin (100 U/ml) and Streptomycin (100 μg/ml) (P06-07050, Pan Biotech, Aidenbach, Germany). HUVECs (cat. no. 00191027, Lonza, Basel, Switzerland) were seeded in HUVEC full medium (cat. no. C-22215 supplemented with C-39215, PromoCell, Heidelberg, Germany) into 0.4 mm ibidi µ-slides (cat. no. 80176, ibidi, Graefelfing, Germany) in a cell density of 0.4–0.5 × 10^6^ cells in 200 µl and allowed to attach for 24 h. When cells were attached, slides were installed into the ibidi pump system (ibidi, Graefelfing, Germany) and HUVECs were cultivated under constant flow (shear stress 5 dyn/cm^2^) for additional seven days, enabling to produce a glycocalyx (Supplementary Fig. 1). To induce an inflammatory environment, both THP-1 cells and HUVECs were stimulated with TNF (5 ng/ml, cat. no. 130-094-022, Miltenyi Biotech, Bergisch Gladbach, Germany) for 16 h before deployment into the flight hardware. Cultivation of both cell lines was performed at 37 °C and 5% CO_2_.

On the experiment day, cells number and viability of THP-1 cells were determined (TC20 cell counter, BioRad, Hercules, CA, USA) and cells were transferred to 1% bovine serum albumin (BSA)/PBS with or without TNF (5 ng/ml) into a 50 ml syringe and kept at 37 °C until installation into the experimental hardware. Ibidi slides containing HUVECs were flushed with 1% BSA/PBS with or without TNF (5 ng/ml) and kept at 37 °C until installation into the experimental hardware. The experimental set-up was performed as described previously^12^.

### Induction of partial gravity by parabolic flight

Experiments were performed during the 81st ESA Parabolic Flight Campaign (17-28 April 2023) at Novespace (Bordeaux, France). On the first and third Parabolic Flight (PF) day, nine parabolas (P0 – 8) were flown in lunar (0.16 *g*, 24 s) gravity, which was followed by seven parabolas in Martian (0.38 *g*, 33 s) gravity, and further seven and eight parabolas in the respective alternating *g*-grade. Each parabola contains a 1.8 *g* hypergravity phase (~25 s for lunar and ~23 s for Martian gravity) at its beginning and end, respectively. One flight day consists of 31 parabolas. The second flight day started with Martian gravity and was continued accordingly in alternating lunar and Martian gravity. To determine the effects of either 0.16 *g* or 0.38 *g*, THP-1 cells and HUVECs were fixated after parabola 8 (P8) for later analysis.

### Experimental run and data recording

Immediately before take-off, syringes with THP-1 cell suspension were installed in the automatic syringe pump device and ibidi slides with HUVECs were mounted on the microscope. After reaching cruising altitude and approximately 10 min before start of the first parabola, pumps were started and cell suspension was pumped into the ibidi flow-chamber. Flow rate was set at 0.4 ml/min, which resulted in a calculated shear stress near the slide surface of 0.05 Pa, which lies in the physiological range of blood flow-induced shear stress in postcapillary venules. A 40x objective subassembly with a CCD camera (Wat-660D, Watec CO., LTD., Yamagata-Ken, Japan) was located near the inlet and recorded passing cells. After passing through the ibidi flow chamber, THP-1 cells were collected and conserved in 15 ml falcon tubes (Thermo Fisher Scientific, Waltham, MA, USA) pre-filled with Transfix (Cytomark, Buckingham, UK). After flight, samples were stored at 4 °C until analysis. After P8, ibidi slides containing HUVECs were removed from the experimental hardware, flushed with PBS and fixated with 2% paraformaldehyde. To avoid over-fixation, samples were flushed again after 10 min with PBS and stored at room temperature until end of flight.

For ground controls, experiments were performed in the same setting under laboratory conditions.

### Determination of floating speed and THP-1 cell adhesion by video analysis

Speed of floating cells in the time frame of P0-8 within different gravitational levels (partial gravity and 1 *g*) was analysed from the video imaging using our Matlab tracking code which extracted and binarized pictures from the videos followed by tracking cells frame by frame of the binary pictures. The floating speed (speed of cell movement adjacent to the substrate) is calculated by the moving distance per time frame and then averaged over the time window passing the frames of vision. Numbers of THP-1 cells attaching to HUVEC cells were determined by counting the number of adherent cells per field of view.

### Assessment of adhesion molecule expression on THP-1 cells by flow cytometry

For adhesion molecule staining, fixated THP-1 cells in falcon tubes were spun down (2500 rpm, 5 min, room temperature) and supernatants were discarded. After two washing steps with PBS pellets were resuspended in 1 ml PBS and 100 µl of cell suspension was transferred in each well of a 96-well plate for antibody staining. Cells were stained in duplicates for 20 min for PSGL-1 (cat. no. 328818), CD44 (cat. no. 397518), LFA-1 (cat. no. 301208) and Mac-1 (cat. no. 301350). All antibodies were obtained from BioLegend (San Diego, CA, USA). Afterwards, samples were washed twice with PBS, resuspended in 200 µl PBS and subsequently analyzed by flow cytometry (Guava® easyCyte™ 8HT Flow Cytometer, Merck Millipore, Billerica, MA, USA). For each measurement, 10,000 events were recorded. Data analysis was performed with InCyte Software for Guava® easyCyte HT Systems (Merck Millipore, Billerica, MA, USA). Representative histograms are depicted in Supplementary Fig. 2.

### Immunocytochemical staining and confocal microscopy

Immediately after flight, the ibidi slides containing fixated HUVEC cells were washed again twice with PBS and subsequently permeabilized with 10% Triton X-100 followed by three times stepwise washing with PBS/0.05% Tween-20 (PBS-T). To avoid unspecific binding, samples were blocked for 10 min with 2% BSA/PBS-T and incubated for two hours with an unconjugated mouse-anti-heparan sulfate antibody (cat. no. 370255-1, Amsbio, Abington, UK). After incubation with this primary antibody, cells were washed three times with PBS-T and subsequently incubated with a secondary fluorochrome-conjugated goat-anti-mouse antibody (cat. no. A11030, Invitrogen, Waltham, MA, USA) as well as with a fluorochrome-conjugated antibodies against ICAM-1 (cat. no. ab214860, Abcam, Cambridge, UK) and with phalloidin (cat. no. A30107, Invitrogen, Waltham, MA, USA) to visualize F-actin. Fluorescence images from the mounted slides were acquired with a Leica TCS SP5 II laser confocal scanning microscope system (Leica, Wetzlar, Germany).

THP-1 cells were counted under LAS X software (Leica, Wetzlar, Germany) according to the morphological characters (roundish with F-actin fiber accumulated around the cell edge). Count and fluorescence intensity of HUVECs were analyzed by ImageJ software (NIH, Bethesda, MD, USA). MFI (mean fluorescence index) of heparan sulfate and ICAM-1 was determined by total fluorescence intensity of respective fluorescence channel divided by cell counts from each high-power field (HPF). F-actin thickness was quantified by measuring the width of concentrated F-actin after deducting background. Quantification of actin filament anisotropy was performed using the FibrilTool as previously reported^13^. Anisotropy index (0–1) indicates the quality of fiber orientation. Value “0” means completely isotropic structure and “1” means completely anisotropic fibril structure, i.e., parallel fibrils. F-actin angles were determined by calculating the angle of the fibers to the right x-axis.

### Computational fluid dynamics (CFD) analysis of cell movement in the device

The flow field in the one flow channels of the device was analyzed using a 2D computational model first built in 0.4 mm $\text{*}$ 6 10 mm by DesginModeler in Ansys Workbench 2023 R1. After creating meshes and defining boundaries, the model was directly imported into FLUENT analytical systems in Ansys Workbench 2023 R1. For a steady flow, the velocity profile within the parallel flow channel can be obtained analytically, given by a parabolic solution of the Poiseuille flow as shown in Eq. (1):

1 $$v\left(y\right)=6\bar{v}\,(h-y)/{h}^{2}$$

Here $\bar{v}$ is the average velocity which is 3.33 mm/s, h is the channel height which is 0.4 mm, and y is the Y coordinate along the height direction. Thus, the inlet velocity profile was set as a parabolic distribution by a user defined file (UDF) to achieve faster convergence. After setting outlet as an outflow boundary, the upper and lower walls were set as still non-slip boundary condition. Then the transient model ran for 600 steps of 0.01 s timestep.

For Discrete Phase Model (DPM), we set the injection as a surface type with 67 randomly distributed particle sources to be consistent with experimental cell density of 10^6^/ml, flow rate of 0.4 ml/min and the timestep of 0.01 s. The diameter of particles was 10 µm and the density of particle was 1.08 g/cm^3^. And the boundary condition for particle was set as elastic by setting the normal and tangential reflection coefficient as 1 except particles would escape in the outlet. The drag force of spherical particle is given by Eqs. (2)–(4):

2 $${F}_{D}=0.5{C}_{D}{\rho }_{f}{A}_{p}{(u-{u}_{p})}^{2}$$

3 $${C}_{D}=24/{R}_{N}({R}_{N} \,<\, 0.1)$$

4 $${R}_{N}={\rho }_{f}{D}_{p}(u-{u}_{p})/\mu$$

Here *C*_*D*_ is coefficient of drag, *ρ*_*f*_ is the density of fluid which equals to 0.998 g/ml, *A*_*p*_ is the reference area of particle which equals to 78.54 µm^2^,*u*_*p*_ and *u* are the velocity of particles and fluid, *R*_*N*_ is the Reynolds number for a spherical particle, *D*_*p*_ is the diameter of the particles, and µ is the viscosity of fluid which equals to 1.003 mPa·s.

### Statistical analysis

Statistical significance tests for multiple groups were performed by two-way ANOVA with Holm-Šídák post Hoc test. *P* < 0.05 was regarded as statistically significant and calculated in SigmaPlot 12.5 (San Jose, CA, USA). Figures were created using Prism 7 (GraphPad Software, La Jolla, CA, USA). All data are presented as mean ± standard deviation (SD). Sample size is indicated in the figure legends.

## Results

### Floating speed under lunar and Martian gravity

**Fig. 1:**
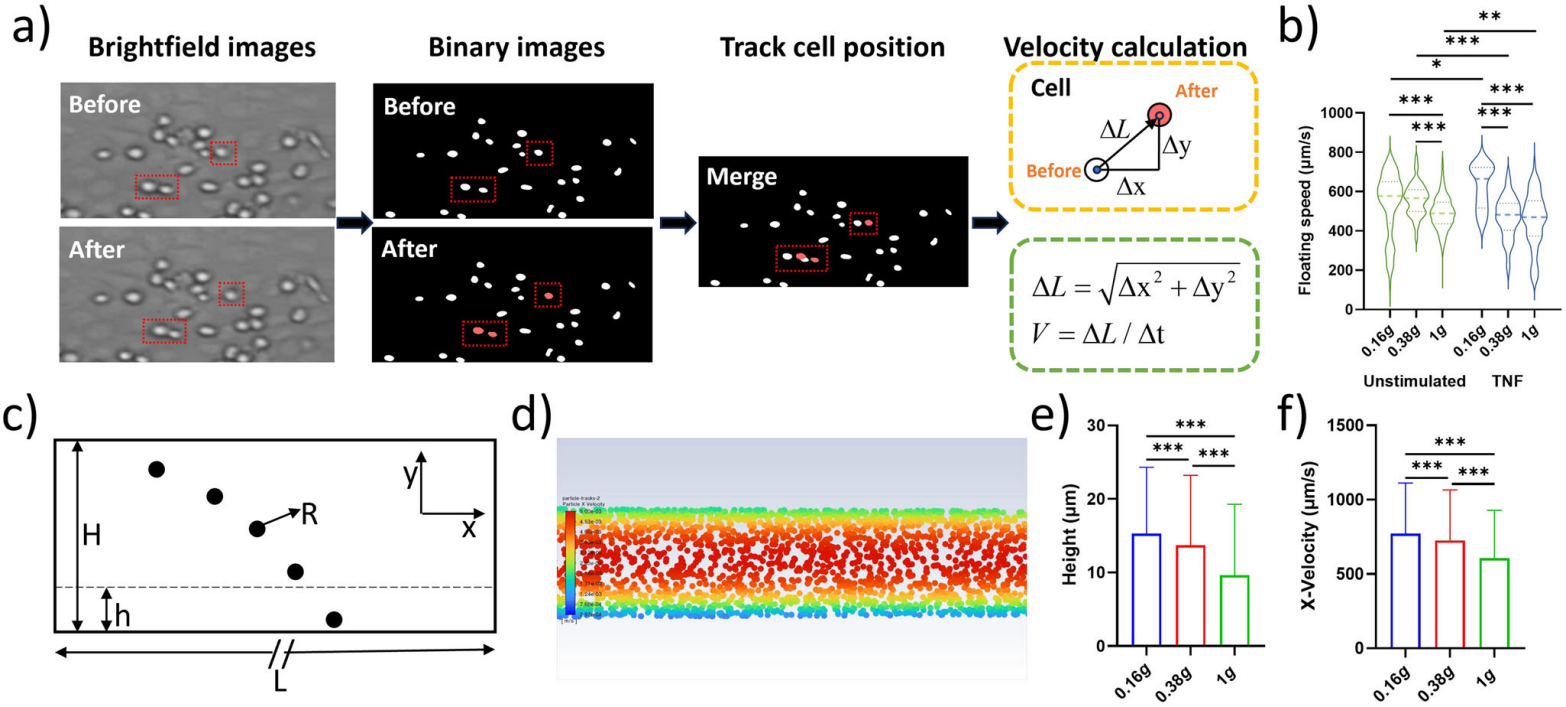
Floating speed in partial gravity. THP-1 cells were injected in a flow chamber coated with a HUVEC cell layer and floating behavior was recorded. Analyzed video sequences derived from multiple parabolas. **a** Flow chart of the image processing and cell tracking. **b** Violine plots representing floating speed (µm/s) of unstimulated cells (left, contoured green) in lunar gravity (0.16 *g*, *n* = 258), Martian gravity (0.38 *g*, *n* = 111) and the 1 *g* control (*n* = 1174) (left side) and TNF-treated cells (5 ng/ml) (right, contoured blue; 0.16 *g*, *n* = 8; 0.38 *g*, *n* = 111; 1 *g*, *n* = 83). Thick dash lines represent mean values and thin dash lines represent quartiles. **c** Schematic of the CFD modeling. **d** The representative image of the cell flowing in the chamber in CFD modeling. **e** Cell height in the boundary zone. **f** Velocity in x-direction of cell in the boundary zone. *P < 0.05, **P < 0.01, ***P < 0.001.

To assess the prerequisite of immune cells to adhere to endothelial vessel walls under partial gravity, the floating speed of THP-1 cells was determined during parabolas in 0.16 *g* and 0.38 *g*. Floating speed during steady flight in 1 *g* served as control. Video analysis (Fig. 1a) revealed that the floating speed of unstimulated THP-1 cells was significantly increased in 0.16 *g* and 0.38 *g* compared to the inflight 1 *g* control. There was no difference in speed between lunar and Martian gravity (Fig. 1b, left side (green plots)). Pre-treatment with TNF led to a significant decrease in floating speed in 0.38 *g* and 1 *g* compared to unstimulated cells, and velocity values at both gravitational levels aligned to the same range (Fig. 1b, right side (blue plots)). TNF-stimulation and 0.16 *g* led to an increase in floating speed compared to 0.38 *g* and 1 *g*, and also to unstimulated cells in 0.16 *g* (Fig. 1b). To explore the increase of floating speed in lunar and Martian gravity, cell movement was additionally modeled the under 0.16 *g*, 0.38 *g* and 1 *g* with DPM of Ansys Fluent (Fig. 1c, d, Supplementary Video 1 (0.16 *g*), 2 (0.38 *g*), 3 (1 *g*)). The x-directional velocity of the cells presented a parabolic profile. We analyzed cells in the boundary zone (area adjacent to the substrate, i.e., h ≤ 30 µm) and found that cells distributed higher under 0.16 *g* and 0.38 *g* than 1 *g* (Fig. 1e) leading to a statistically larger x-directional velocity (floating speed) under 0.16 *g* and 0.38 *g* than in 1 *g* (Fig. 1f).

### Cell adhesion to HUVEC layer

**Fig. 2:**
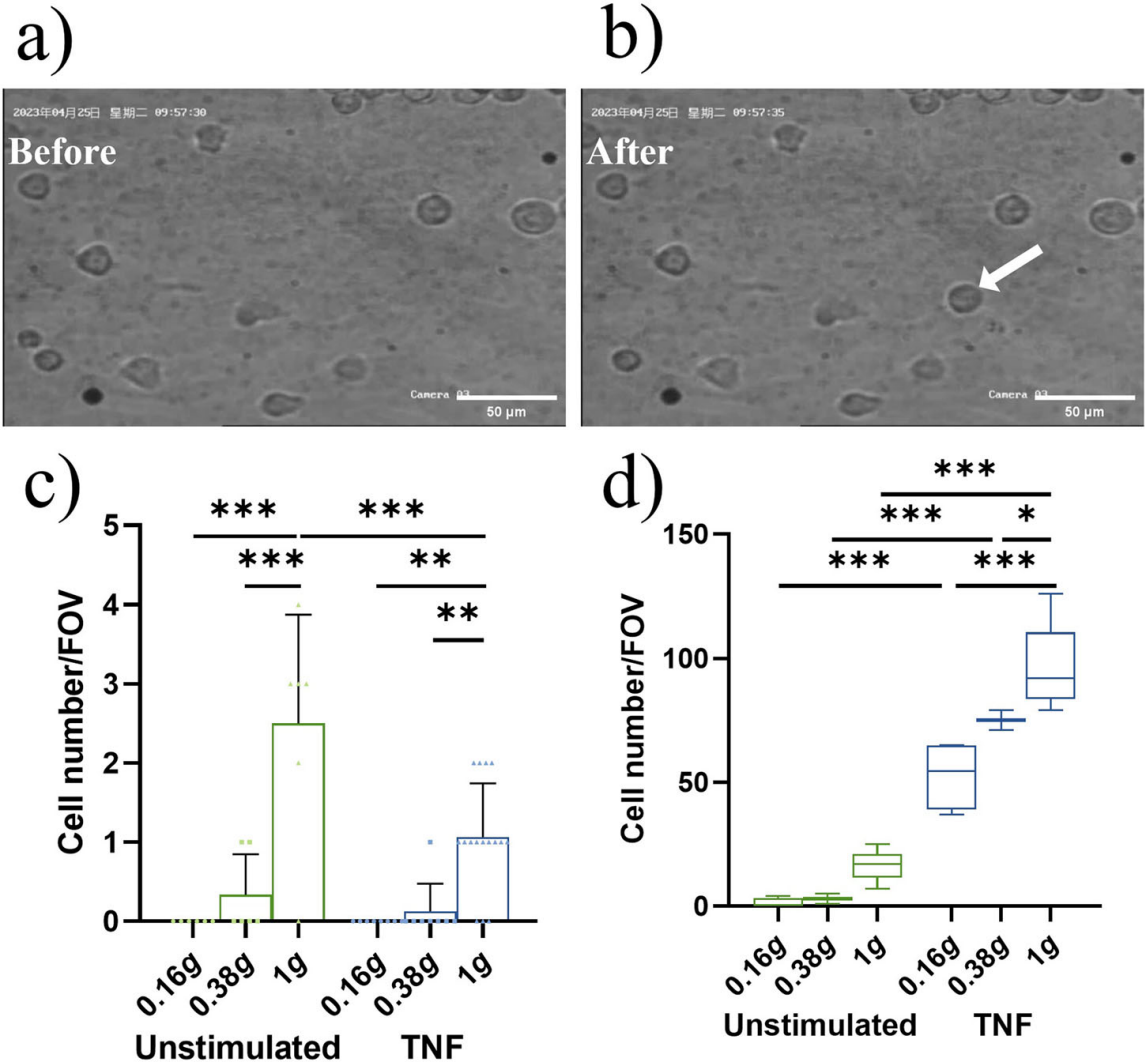
THP-1 cell adhesion to HUVEC layer. Representative images (**a**) before and (**b**) after floating THP-1 cells adhere to the HUVEC layer (arrow). Scale bar: 50 µm. **c** The number of newly adhering THP-1 cells to the HUVEC layer within each parabola of the partial gravity phases (0.16 *g* and 0.38 *g*) and in 1 *g* assessed by video analysis (cell number/FOV (field of view)). Bar charts represent mean values ± SD and symbols represent number of cells/FOV. **d** Total number of THP-1 cells adhering to HUVEC layer assessed by confocal microscopic assessment (cell number/FOV). Inflight fixation of samples was performed after P8 and numbers were compared to baseline collection on ground (1 *g*). Boxes indicate median and interquartile range; whiskers represent minimum and maximum (three FOV/slide; two slides per condition for 0.16 *g* and 1 *g* and one slide per condition for 0.38 *g*). *P < 0.05, **P < 0.01, ***P < 0.001.

The increase in floating speed of unstimulated THP-1 cells in 0.16 *g* and 0.38 *g* in the videos was accompanied by reduced cell adhesion to the HUVEC cell layer (Fig. 2a, b) in comparison to the inflight 1 *g* control (Fig. 2c). Interestingly, but in discordance with the reduced floating speed of TNF-treated THP-1 cells in 0.38 *g* and 1 *g*, the number of newly attached cells per visual field was remarkably lower in TNF-stimulated cells than in unstimulated cells (Fig. 2c). This counterintuitive phenomenon might be due to a generally higher number of attached cells by TNF treatment, which however could have led to a capturing of the THP-1 cells outside of the visual field for video analysis. This conclusion was supported by immunocytochemical staining of THP-1 cells within the slides that were fixated after completion of P8 and analyzed by confocal fluorescence microscopy and different visual fields. The number of cells sticking to the HUVEC layer was significantly higher in the TNF-treated samples compared to the unstimulated ones. Regarding the pure effects of 0.16 *g* or 0.38 *g*, the number of adhering THP-1 cells was reduced with decreasing *g*-grades (Fig. 2d).

### Cell-cell interactions in partial gravity

To investigate whether the altered interaction levels between THP-1 cells and the HUVEC layer in partial gravity might be due to differentially expressed adhesion molecules, surface expression levels of PSGL-1, LFA1, Mac-1 and CD44 were assessed on THP-1 cells after passing the flow chamber and ICAM-1 expression was determined on HUVECs. Inflight samples which were not assembled into the flow chamber system and fixated before start of parabolas served as controls.

The expression of Mac-1 was enhanced on THP-1 cells after passage through the HUVEC-coated flow chamber in 0.16 *g*, which reached statistical significance for untreated cells. A single stimulation with TNF had no effect on Mac-1 expression (Table 1). THP-1 control cells, which were flown outside of the flow chamber system and fixated after P8 displayed Mac-1 levels that were comparable to the pre-parabola inflight controls (Supplementary Table 1), indicating that the upregulation of this adhesion molecule is exclusive to flow chamber conditions.

**Table 1:** Expression (MFI) of Mac-1 on THP-1 cells inflight before start of parabolas (1 *g*) and after completion of P8 in lunar gravity (0.16 *g*)

*Mac-1*	inflight control (1 *g*)	lunar gravity (0.16 *g*)
unstimulated	20.41 ± 3.79	**28.62** ± 4.18**
TNF (5 ng/ml)	19.14 ± 0.33	30.80 ± 3.42

Data show mean ± STD, *n* = 2

*significant difference between expression in cells in 1 *g* and 0.16 *g* (**P < 0.01)

The expression levels of the adhesion molecules PSGL-1 and CD44 were neither affected by TNF stimulation, nor by partial gravity (Supplementary Table 1). LFA-1 levels were lower after passing the flow chamber in 0.16 *g*, especially on TNF treated cells. However, inter-experimental variations were quite pronounced for this surface molecule and values failed to reach statistical significance.

**Fig. 3:**
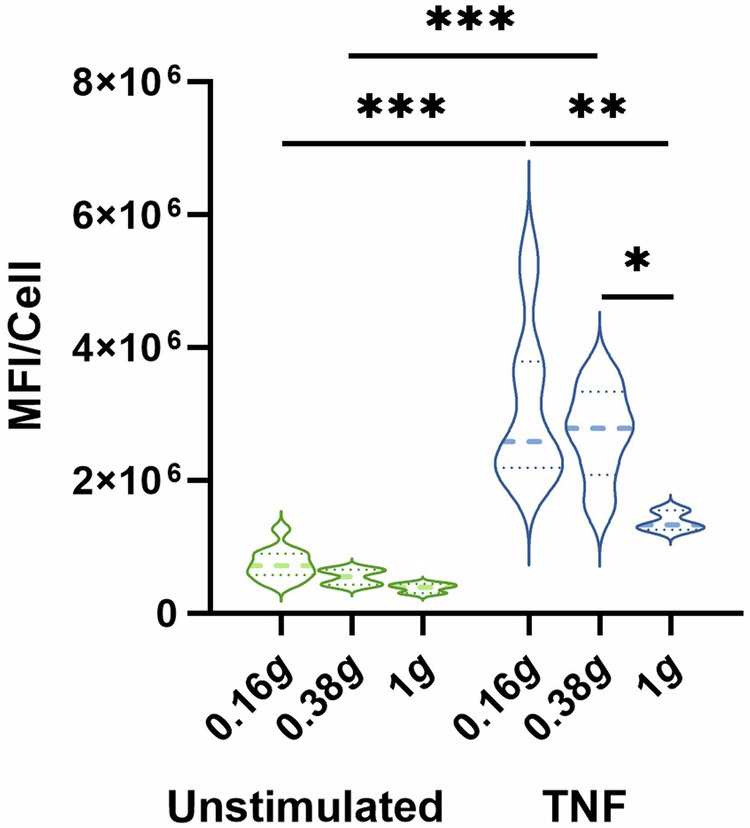
ICAM-1 expression on HUVEC cells. Average MFI/cell of ICAM-1 expression on and in HUVEC cells. Fixation of cells was performed either before start of parabolas (inflight control without flow, 1 *g*) or after P8 in Martian or lunar gravity, respectively. Thick dash lines represent mean values and thin dash lines represent quartiles. *P < 0.05, **P < 0.01, ***P < 0.001 (*n* = 3–12).

In HUVECs, TNF stimulation in 1 *g* induced an increased expression of ICAM-1. Exposure to 0.38 *g* and 0.16 *g* resulted in a gradual increase in ICAM-1 expression and this was further augmented by TNF stimulation (Fig. 3).

### Effect of partial gravity on F-actin organization in HUVECs

**Fig. 4:**
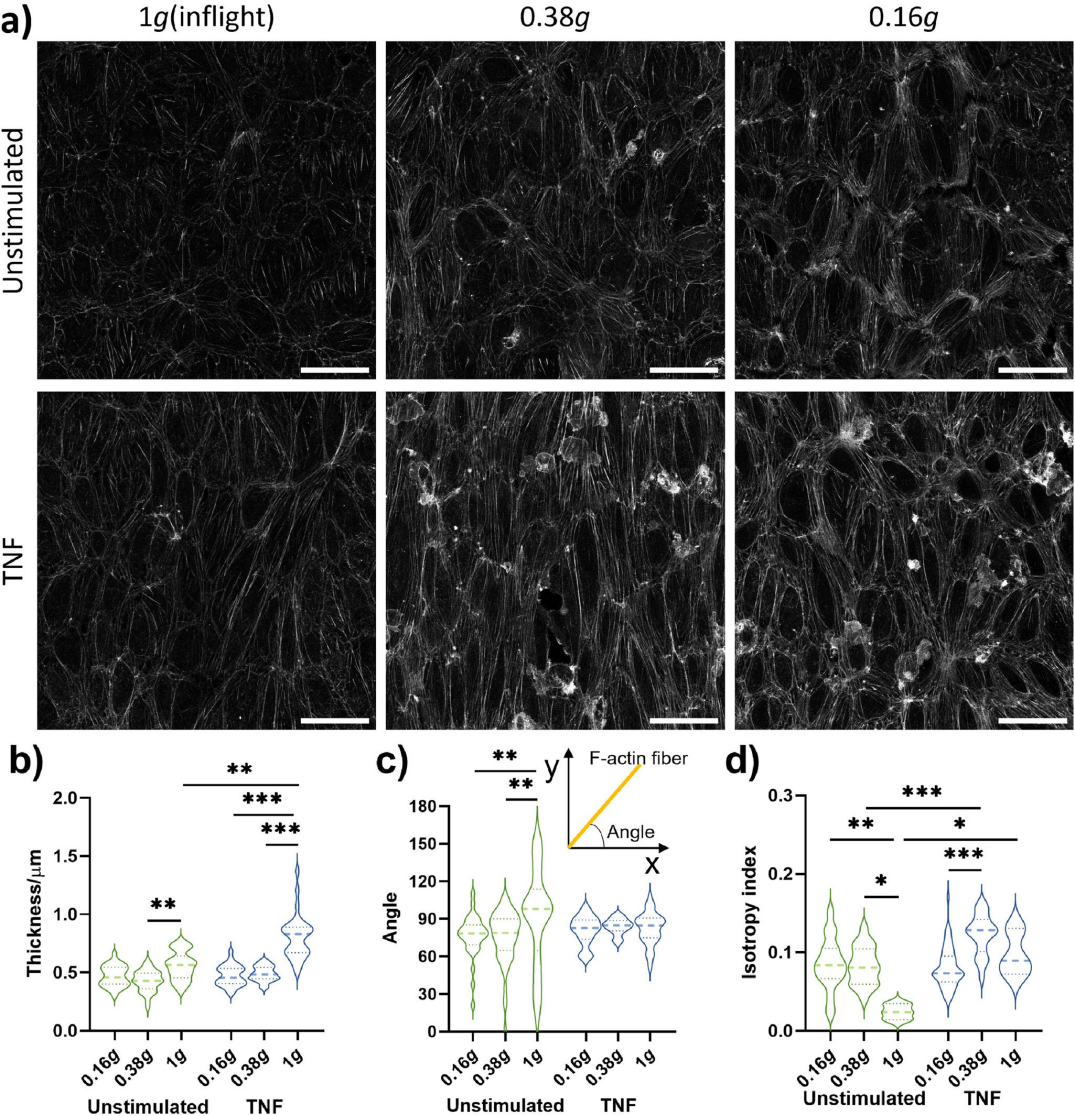
F-actin organization in HUVEC cells. Fixation of cells was performed either before start of parabolas (inflight control without flow, 1 *g*) or after eight parabolas in lunar (0.16 *g*) or Martian (0.38 *g*) gravity, respectively. **a** Overview of representative images of F-Actin by confocal microscopy. Roundish structures on the F-actin network are adherent THP-1 cells. **b** thickness, (**c**) angle, (**d**) anisotropy of F-actin. Thick dash lines in violin plots represent mean values and thin dash lines represent quartiles. *P < .05, **P < 0.01, ***P < 0.001 (*n* = 1 for 0.38 *g* and *n* = 2 for 0.16 *g*; three FOVs per slide analyzed).

Stress fibers are highly coordinated cytoskeleton structures composed of actin filament bundles and often myosin, which plays a fundamental role in cellular processes such as adhesion and morphogenesis. Stress fibers constantly undergo dynamic assembly and disassembly to maintain cellular tension and respond to various mechanical forces. Thus, we examined changes in F-actin organization to assess the impact of partial gravity on the endothelial cytoskeleton. TNF-stimulation alone could induce enhanced stress fiber formation and cell elongation (Fig. 4a, left column). Compared to unstimulated cells, stimulation with TNF induced an enhanced stress fiber thickness (Fig. 4b) as well as a more coordinated alignment which was demonstrated by condensed fiber angles (Fig. 4c) and a higher fiber anisotropy index (Fig. 4d), indicating an increased directionality of these HUVECs under TNF stimulation. The amount of microfilament networks decreased visually when HUVECs were exposed to 0.38 *g* and 0.16 *g* (Fig. 4a, middle and right column) and actin stress fiber bundles aligned to cell borders. Stress fiber thickness was remarkably reduced in partial gravity compared to the inflight control with significant differences in TNF-treated samples (Fig. 4b). In unstimulated HUVECs, fiber angles (Fig. 4c, insert) were more condensed in partial gravity compared to the inflight control, while cells from all TNF-treated approaches showed strongly condensed angles (Fig. 4c). In 0.38 *g*, anisotropy index was increased by TNF stimulation but this effect completely disappeared in 0.16 *g*. In summary, confocal microscopy analysis of F-actin demonstrated an enhanced directionality of HUVEC cytoskeleton by TNF and by partial gravity, and the alterations in directionality by partial gravity was more profound under unstimulated conditions compared to TNF stimulation.

## Discussion

The realization of the endeavor to land humans on the moon again or to travel even one day to Mars requires a preceding clarification of the impact of deep space explorations on human physiology. Among the most prominent factors are long-term isolation, radiation, and partial gravity, which amounts for 1.62 m/s^2^ (0.16 *g*) on the moon and 3.69 m/s^2^ (0.38 *g*) on Mars. Knowledge about risks of reduced gravity on human health derives by now from research in real and simulated microgravity (µ*g*). In view of immunology, µ*g* was characterized to be among the main factors causing dysregulations^14^. Monocytes show a subclinical, however increased pro-inflammatory potential in µ*g*^11,15^ while oxidative burst and phagocytosis capacities are impaired both in monocytes/macrophages and in granulocytes^16^. T cells may express either reduced activation capacities in response to antigenic stimulation or proneness to autoimmunological reactions^17,18^. Altogether, these dysregulations may induce premature immune aging and cause damage to astronauts´ health^16,19^. Moreover, in previous parabolic flight experiments, we observed an increased rolling speed and reduced rolling rate of PBMCs on an adhesion molecules substrate in a flow chamber setting in µ*g*, which may inhibit leukodiapedesis^12^. We proposed prevailing lift forces and a consequent centralization of flow in µ*g* to impede contact between suspension cells and the adhesion molecule substrate^12,20^. In the present study we translated our flow chamber setting to lunar (0.16 *g*) and Martian (0.38 *g*) gravity, which was to our knowledge the first experimental set-up to investigate immune cell behavior in partial gravity. In addition, we replaced the initial adhesion molecule coating with cultivation of HUVECs under seven days of constant flow, which induced the cells to produce an endothelial glycocalyx (Supplementary Fig. 1), thus optimizing the simulated vessel endothelium. A glycocalyx layers the luminal surface of the vascular endothelium, thereby providing structural support and vascular integrity^21–23^. Moreover, the glycocalyx is involved in inflammatory processes by containing adhesion molecules such as ICAM-1, which protrude from endothelial cells^21,24,25^ and mediate leukocyte binding. However, for inflammatory regulation, the predominant parts of these adhesion molecules are shielded by the glycocalyx and exposed only on demand by glycocalyx degradation^25^.

Comparable to our µ*g* setting, we determined an increased floating speed of THP-1 cells in partial gravity, which we attributed to a centralization of particles mediated by reduced gravitational forces as indicated by CFD simulations. This increase in floating speed was similar in 0.16 *g* and 0.38 *g* when cells were unstimulated. When both HUVECs and THP-1 cells were pre-treated with TNF, floating speed was still increased in 0.16 *g* while in 0.38 *g*, values aligned to 1 *g* levels. This suggests a certain degree of gradual effect, which was—in this experimental setting – only noticeably pronounced under a TNF-induced inflammatory environment. We propose an involvement of secreted mediators by HUVECs^26^, such as cytokines and chemokines, which orchestrate THP-1 cells to the HUVEC layer. In addition, TNF may induce an onset of degradation of the glycocalyx, leading to a full presentation of adhesion molecules and thereby facilitating leukocyte binding^25,27^. However, due to the experimental set-up and boundary conditions in this study, it was not possible to collect the flow-through of the flow chambers for analysis of secreted mediators such as cytokines and chemokines as well as of glycocalyx components which would represent a sign for glycocalyx degradation.

Because of the weaker lift forces in 0.38 *g*, cells were able to encounter centralization. This assumption is supported by the reduced attachment of THP-1 cells in dependence of *g*-grade and a rising adhesion molecule expression both on HUVECs and THP-1 cells from normogravity (1 *g*) over Martian to lunar gravity, which of note occurred already early in parabolic flight. Analyses of fixated cells after P8 revealed an increased expression of the β2 integrin Mac-1 on THP-1 cells and of the immunoglobulin superfamily member ICAM-1 on HUVECs, which are known to interact with each other^28^ and to belong to the initiating steps of transendothelial migration^29^. In THP-1 cells, the increase in Mac-1 levels occurred after passage through the flow chamber. Since such an increase in Mac-1 expression levels didn´t occur in control cells outside of the flow chamber setting, neither by TNF, nor by exposure with partial gravity or a combination of both (Supplementary Table 1), we assume that the secretory and potentially pro-inflammatory environment produced by HUVECs in the flow chamber induced this increase of expression to enhance the possibility of a firm binding. A pro-inflammatory environment has been shown to increase Mac-1 expression^30^, which suggests that a single TNF stimulation or the concentration used in our experimental setting could have been insufficient to enhance Mac-1 expression levels. If our opposite observation of lower LFA-1 levels in 0.16 *g* can be declared as experimental variations or if this adhesion molecule might have been downregulated in response to partial gravity conditions represents a matter of future investigations. Here it has to be noted that, because of the flight schedule of the present parabolic flight campaign, only two sets of experiments could be collected for lunar gravity (flight day one and three; flight day two in Martian gravity until P8). On HUVECs, expression of ICAM-1 was already increased by single treatment with TNF and exposure to partial gravity. A combination of both partial gravity and TNF resulted in a more than additive augmentation of ICAM-1 expression. The effect of simulated µ*g* on the expression of the adhesion molecules E-selectin, ICAM-1, VCAM-1 and VE-cadherin was investigated by Buravkova and colleagues^31^. They have demonstrated an increased expression of these adhesion molecules after 24 h exposure to simulated µ*g* and this was exacerbated by TNF. However, the observed effects were dependent of the present activation state of the endothelial cells^31^. Thus, our findings and the observations of Buravkova et al. indicate an enhanced expression of adhesion molecules on activated endothelial cells in reduced gravity which may, when immune cells come into contact with endothelial cells, foster transendothelial migration and inflammation. In addition to an altered adhesion behavior^32^, a differential adhesion molecule expression on the endothelial cell surface might induce cytological changes^33^.

Analysis of F-actin displayed a spindle-like shape of HUVECs, which is primarily attributed to the culturing conditions. Prior to the PF experiment, cells were cultured for 7 days under constant flow and a shear stress of 5 dyn/cm^2^, which leads to an alignment of the cells along their long axis to the direction of flow and the rearrangement of F-actin into bundles of stress fibers^34^. Pretreatment with TNF further intensified stress fiber formation and cell elongation which was also reported by others^35,36^. Exposure to partial gravity further added to the effect on the cell culture-mediated cytoskeletal reorganization in HUVECs. Stress fibers aligned even more to cell borders with a concomitant actin filament thinning as it has been previously described^37–39^. This phenomenon was observed both under real and simulated µ*g* after short- (1–2 h)^34,39^ and long-term (24 h and longer)^34,37,38^ exposure. Such changes in cell morphology might affect permeability and barrier functions of endothelial cells and induce changes in inflammatory processes^34,40^.

Altogether, however, it shall be mentioned that the observed effects of partial gravity on adhesion molecule expression and cytoskeletal reorganization may also include effects caused by the inevitable hypergravity (1.8 *g*) phases which occur both at the beginning and at the end of each parabola.

In conclusion, this is to our knowledge the first study that investigated immune cell flow, adhesion to an endothelial layer as well as cytoskeletal changes under real partial gravity in an acute short-term model. It appears that the impacts on cell flow, adhesion marker expression, and F-actin reorganization are comparable to findings that were made in µ*g*. In lunar gravity (0.16 *g*) alterations seem to be more pronounced than in Martian gravity (0.38 *g*). For investigations of gradual effects, PF flight schedules with profiles reflecting phases of µ*g*, lunar and Martian gravity shall be further applied. To investigate the impact of partial gravity on immune cells and their interaction with a vascular endothelium in greater detail, analyses in short- and long-term simulated partial gravity settings are required.

## Supplementary information

Supplementary information

Supplementary Video 1

Supplementary Video 2

Supplementary Video 3

## Data Availability

Supplementary information accompanies this manuscript and is attached as single file.
