# Supplementary material for: Lunar and Martian gravity alter immune cell interactions with endothelia in parabolic flight
Source: NPJ Microgravity. 2025 Feb 3;11:4. doi: 10.1038/s41526-024-00456-7 (PMC11791073; doi:10.1038/s41526-024-00456-7)
Supplement: Supplementary file 1 — Supplementary information [file 41526_2024_456_MOESM1_ESM.pdf]

**Supplementary information for**

# Lunar and Martian gravity alter immune cell interactions with endothelia in parabolic flight

Yu Du, Bing Han, Katharina Biere, Nathalie Abdelmalek, Xinyu Shu, Chaoyang Song, Guangyao Chen, Ning Li, Marina Tuschen, Huan Wu, Shujin Sun, Alexander Choukér, Mian Long, Dominique Moser

Correspondence to
Alexander Choukér: achouker@med.uni-muenchen.de
Mian Long: mlong@imech.ac.cn

This file includes:
Supplementary Figure 1
Supplementary Figure 2
Supplementary Table 1

Separate video files:
Supplementary video 1
Supplementary video 2
Supplementary video 3

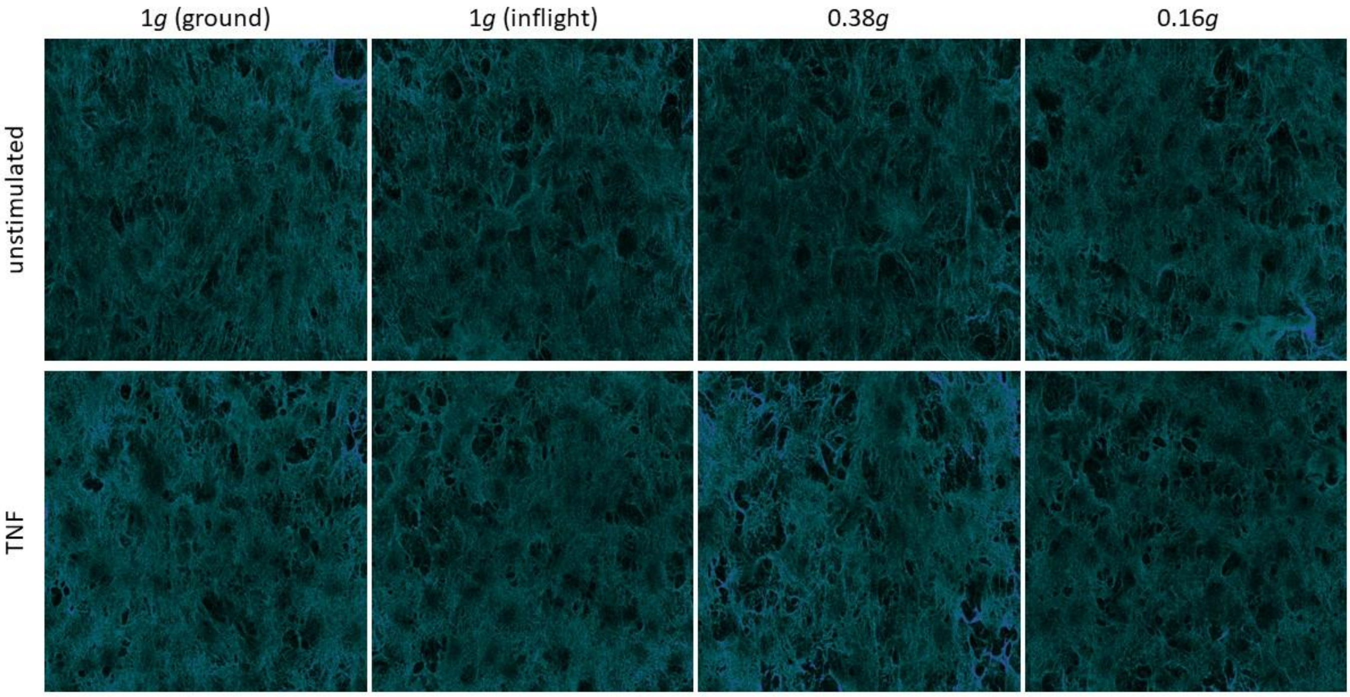


**Supplementary Figure 1 Expression of glycocalyx on HUVECs.** Fixation of cells was performed either on ground at the time of start of parabolas (1*g* (ground)), in the plane before start of parabolas (1*g* (inflight)), or after eight parabolas in Martian (0.38*g*) or lunar (0.16*g*) gravity. Representative images of the glycocalyx component heparan sulfate by confocal microscopy.

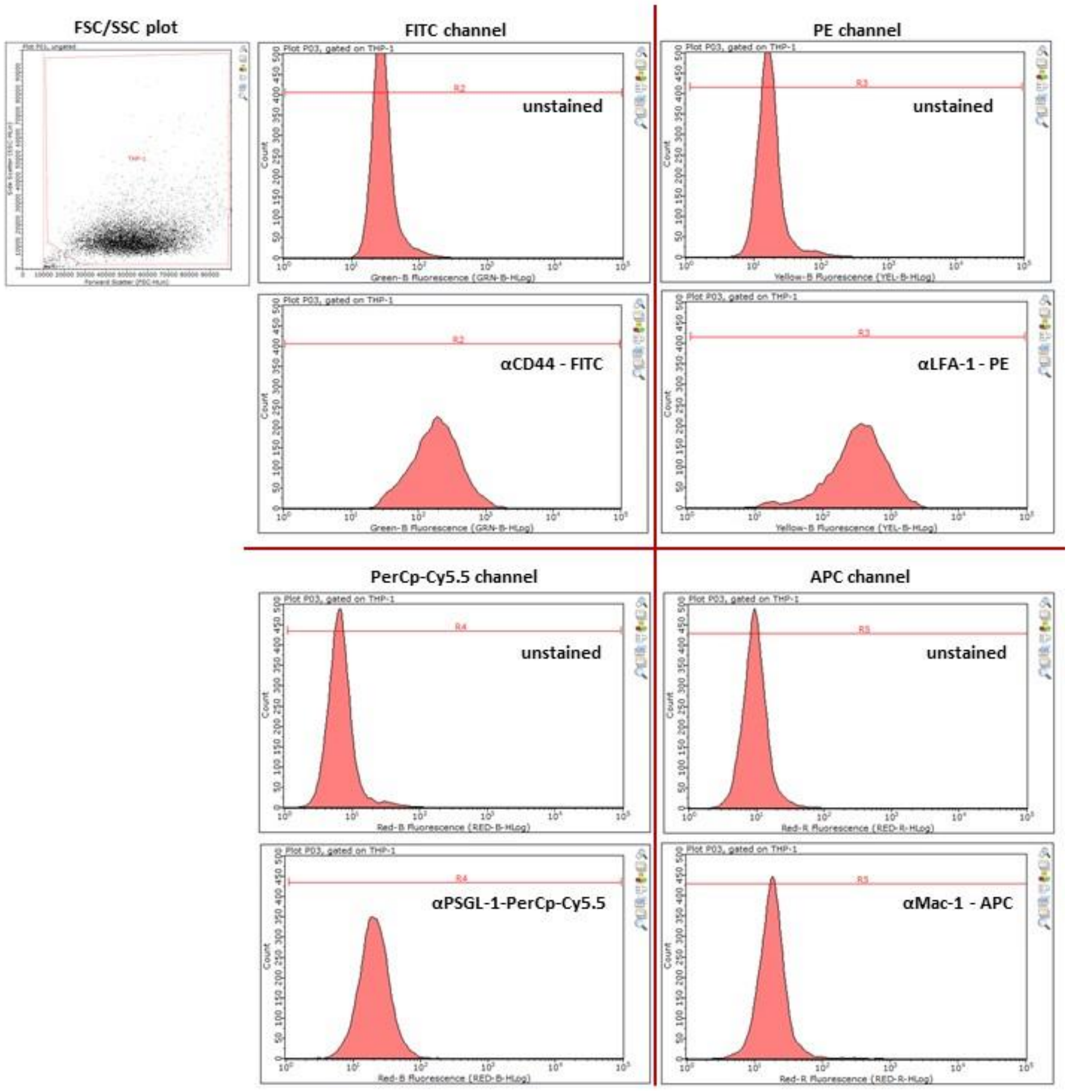


**Supplementary Figure 2 Representative flow cytometry histograms.** FSC/SSC plot of THP-1 cells and histograms showing intensity of antibody binding in comparison to their respective negative control. Upper left: αCD44-FITC, upper right: αLFA-1-PE, lower left: αPSGL-1-PerCp-Cy5.5, lower right: αMac-1-APC.

**Supplementary Table 1 Surface adhesion marker expression on THP-1 cells.** Expression (MFI) of Mac-1, PSGL-1, CD44 and LFA-1 on THP-1 cells inflight before start of parabolas (1*g*) and after completion of P8 in lunar gravity (0.16*g*). Data show mean ± STD, n=2, **significant difference between expression on cells in 0.16*g* and different 1*g* control conditions (** *P*<0.01).

| | **Ground control** | | **Inflight - pre-parabola** | | **Inflight - after P8** | | **Inflight - 0.16*g* (flow chamber)** | |
|---|---|---|---|---|---|---|---|---|
| | unst. | TNF | unst. | TNF | unst. | TNF | unst. | TNF |
| **Mac-1** | **17.26 ± 2.46**** | 21.71 ± 7.12 | **20.41 ± 3.79**** | 19.14 ± 0.33 | **16.56 ± 1.10**** | 19.25 ± 1.68 | 28.62 ± 4.18 | 30.80 ± 3.42 |
| **PSGL-1** | 23.30 ± 0.64 | 20.32 ± 0.23 | 22.30 ± 0.09 | 21.16 ± 0.67 | 23.41 ± 1.24 | 21.85 ± 3.39 | 26.26 ± 2.10 | 23.14 ± 1.57 |
| **CD44** | 166.37 ± 7.11 | 185.22 ± 34.83 | 162.08 ± 8.02 | 176.41 ± 8.41 | 160.71± 14.95 | 184.36± 69.33 | 151.05 ± 38.37 | 142.36± 25.27 |
| **LFA-1** | 370.66 ± 29.82 | 370.27 ± 14.81 | 323.18 ± 46.46 | 333.60 ± 30.60 | 352.64 ± 67.39 | 381.54 ± 118.52 | 322.56 ± 144.69 | 254.97 ± 59.09 |

## Figure legends Supplementary Videos

**Supplementary Video 1 Computational fluid dynamics in lunar gravity** Modeling of cell movement in 0.16*g* with Discrete Phase Model of Ansys Fluent.

**Supplementary Video 2 Computational fluid dynamics in Martian gravity** Modeling of cell movement in 0.38*g* with Discrete Phase Model of Ansys Fluent.

**Supplementary Video 3 Computational fluid dynamics in normogravity** Modeling of cell movement in 1*g* with Discrete Phase Model of Ansys Fluent.
